# Implication of Abscisic Acid on Ripening and Quality in Sweet Cherries: Differential Effects during Pre- and Post-harvest

**DOI:** 10.3389/fpls.2016.00602

**Published:** 2016-05-04

**Authors:** Verónica Tijero, Natalia Teribia, Paula Muñoz, Sergi Munné-Bosch

**Affiliations:** Department of Plant Biology, Faculty of Biology, University of BarcelonaBarcelona, Spain

**Keywords:** sweet cherry, ABA, ripening, over-ripening, ascorbate, vitamin E, cold storage

## Abstract

Sweet cherry, a non-climacteric fruit, is usually cold-stored during post-harvest to prevent over-ripening. The aim of the study was to evaluate the role of abscisic acid (ABA) on fruit growth and ripening of this fruit, considering as well its putative implication in over-ripening and effects on quality. We measured the endogenous concentrations of ABA during the ripening of sweet cherries (*Prunus avium* L. var. Prime Giant) collected from orchard trees and in cherries exposed to 4°C and 23°C during 10 days of post-harvest. Furthermore, we examined to what extent endogenous ABA concentrations were related to quality parameters, such as fruit biomass, anthocyanin accumulation and levels of vitamins C and E. Endogenous concentrations of ABA in fruits increased progressively during fruit growth and ripening on the tree, to decrease later during post-harvest at 23°C. Cold treatment, however, increased ABA levels and led to an inhibition of over-ripening. Furthermore, ABA levels positively correlated with anthocyanin and vitamin E levels during pre-harvest, but not during post-harvest. We conclude that ABA plays a major role in sweet cherry development, stimulating its ripening process and positively influencing quality parameters during pre-harvest. The possible influence of ABA preventing over-ripening in cold-stored sweet cherries is also discussed.

## Introduction

In recent decades, sweet cherry has become one of the most important non-climacteric fruits worldwide, with an important distribution to international markets from highly productive countries at origin, such as Turkey, United States, Iran, Italy, and Spain, among others (FAO, 2015). However, both its flavor and nutritional quality is strongly dependent on tree growth conditions at pre-harvest, post-harvest treatments and its consumption at an optimum ripening stage (Ashton, 2007). Over-ripening, which leads to a loss of quality during post-harvest, is associated with fruit darkening, softening and a general loss of organoleptic properties (Meheriuk et al., 1995). Cold treatments are generally used to store them properly during post-harvest in order to avoid fruit quality loss, but the physiological and biochemical mechanisms underlying fruit ripening on the tree and over-ripening during post-harvest are still relatively unknown for sweet cherries.

It has been shown that high concentrations of abscisic acid (ABA) are required for ripening in sweet cherries (Luo et al., 2013; Wang et al., 2015). ABA is a sesquiterpenoid hormone, derived from carotenoids, that is implicated in several physiological processes, from seed dormancy to senescence processes, including plant stress responses and the regulation of fruit development (Nambara and Marion-Poll, 2005; Finkelstein, 2013; Leng et al., 2014). ABA has been shown to play a major role in the ripening process of non-climacteric fleshy fruits, such as cherry fruits, modulating color changes (through modulation of anthocyanin biosynthesis) and sugar accumulation (Kumar et al., 2014; Wang et al., 2015). However, nothing is known about the possible role of ABA in the regulation of fruit quality in terms of vitamin C and E accumulation, or to what extent ABA can affect over-ripening processes in sweet cherries.

Among various quality parameters, the content and composition of water- and lipid-soluble vitamins in edible fleshy fruits is of paramount importance for human health (FAO, 2004). Sweet cherries are rich in vitamin C, which is considered one of the most important water-soluble antioxidants, together with anthocyanins, in this fruit (Serrano et al., 2005). Aside from protecting cells from reactive oxygen species, ascorbate is involved in the regulation of growth processes in plants (Veljovic-Jovanovic et al., 2001), and it plays a role, as a cofactor, in the regulation of 9-*cis*-epoxycarotenoid dioxygenase (NCED), the key limiting step in the biosynthesis of ABA from carotenoids (Conklin and Barth, 2004). Furthermore, ascorbate recycles oxidized tocopherols (vitamin E), when this lipid-soluble antioxidant reacts with lipid peroxyl radicals in its function of inhibiting the propagation of lipid peroxidation in biological membranes (Munné-Bosch and Alegre, 2002). Although vitamin C has received some attention in the ripening of sweet cherries as a component of organic acids (Serrano et al., 2005), nothing is known about the levels of vitamin E, its possible variations with ripening and regulation by phytohormones in non-climacteric fruits. Only in mango, a climacteric fruit, it has been shown that vitamin E biosynthesis may be modulated by ethylene (Singh et al., 2011).

The aim of this study was to get some insights into the role of ABA in the ripening process of sweet cherries, focusing on the endogenous levels of this phytohormone during fruit development in orchard trees and under different conditions of post-harvest. In addition, to better understand the role of ABA in ripening, as well as the loss of quality during fruit storage, we simultaneously analyzed various parameters associated with the ripening process and the fruit quality, such as fruit biomass, anthocyanin accumulation, and levels of antioxidants, including carotenoids, and vitamins C and E.

## Materials and Methods

### Experimental Design and Sampling

Three independent, complementary experiments were performed using sweet cherries (*Prunus avium* L. *var.* Prime Giant). The first experiment focused on a study of fruit ripening on the tree followed by an over-ripening process at 23°C, the second one was performed preventing over-ripening at 4°C, and the third one was performed to test for the tissular location of vitamins in cherry fruits.

For the first experiment, sweet cherries were obtained from trees growing in an exploited orchard at Partida Vall del Sector III (Lleida, NE Spain). Fruits were harvested at various developmental stages on the tree between 23 and 4 days before harvest, and between 3 and 10 days of post-harvest at 23°C, which led to over-ripening (Supplementary Figure S1). First sampling in orchard trees was performed during 30th April 2015 (23 days before harvest), which corresponds to 34 days after full bloom. For the second experiment, 10 kg from the same cherry cultivar and orchard were brought to the laboratory 3 days after commercial harvest. Fruits without visual defects were chosen for experiments. Then, half of the fruits were kept at 23 ± 2°C in the laboratory, while the other half were subject to 4 ± 1°C in a cold chamber. In both cases, fruits were kept in darkness and samples were taken daily during storage for 1 week.

A third experiment was performed to evaluate possible tissue-specific accumulation of vitamins in sweet cherries. The pit, flesh and skin from fruits collected 23 days pre-harvest or 3 days post-harvest were manually separated and immediately immersed in liquid nitrogen for hormone, anthocyanin and vitamins C and E analyses.

All samplings were performed early in the morning (between 9 and 10 a.m. local time) with an average temperature of 10 ± 2°C during pre-harvest and 23 ± 2°C during post-harvest for the first experiment, and with an average temperature of 4 ± 1°C for the second one. Six fruits per tree from eight trees were randomly sampled at each time point during pre-harvest, and six fruits from commercial boxes were randomly sampled daily during post-harvest, for each, 23°C and cold storage. For all experiments, samples were immediately snap frozen in liquid nitrogen and stored at -80°C until analyses.

### Endogenous Concentrations of Abscisic Acid

Abscisic acid levels were determined by ultrahigh-performance liquid chromatography coupled to tandem mass spectrometry (UHPLC-MS/MS) as described previously (Müller and Munné-Bosch, 2011). In short, 100 mg per sample were extracted with 200 μL methanol:isopropanol:acetic acid 50:49:1 (v/v/v) using ultrasonication and vortexing (Branson 2510 ultrasonic cleaner, Bransonic, Danbury, CT, USA) for 30 min. Deuterium-labeled ABA was then added, and after centrifugation at 600 *g* for 15 min at 4°C, the pellet was re-extracted using the same procedure. Supernatants were pooled and filtered through a 0.22 μm PTFE filter (Waters, Milford, MA, USA) before analyses. ABA levels were analyzed by using UHPLC-ESI-MS/MS as described in Müller and Munné-Bosch (2011). Quantification was made considering recovery rates for each sample by using a deuterium-labeled internal standard.

### Fruit Quality Parameters

Fruit biomass was estimated by weighing the samples immediately at each sampling time point or after transferring them to the laboratory in bags (with high humidity to avoid desiccation).

Total anthocyanins were determined spectrophotometrically in methanolic extracts as described (Gitelson et al., 2001). In short, 200 mg per sample were extracted with 1 mL methanol using ultrasonication and vortexing. Extracts were centrifuged at 600 *g* for 10 min at 4°C and the pellet was re-extracted following the same procedure. Supernatants were pooled and 1% HCl was added. Then, total anthocyanins were measured spectrophotometrically at 530 nm. Total anthocyanins were calculated using the molar extinction coefficient of cyanidin-3-glucoside as a reference, as described (Siegelman and Hendricks, 1958).

Carotenoids levels were estimated by HPLC after extraction with methanol, as described (Munné-Bosch and Alegre, 2000). In short, samples were extracted with methanol, as described for anthocyanins, and separated on a Dupont non-endcapped Zorbax ODS-5 μm column (250 mm long, 4.6 mm i.d.; 20% Carbon, Teknokroma, St. Cugat, Spain) at 30°C for 38 min at a flow rate of 1 mL min^-1^. The solvent mixture for the gradient consisted of (A) acetonitrile:methanol (85:15, v/v) and (B) methanol:ethyl acetate (68:32, v/v). The gradient used was: 0–14 min 100% A, 0% B; 14–16 min decreasing to 0% A, 100% B; 16–28 min 0% A, 100% B; 28–30 min increasing to 100% A, 0% B; and 30–38 min 100% A, 0% B. Detection was carried out at 445 nm and compounds were identified and quantified as described previously (Munné-Bosch and Alegre, 2000).

The analysis of vitamin C was adapted from Takahama and Oniki (1992) and Queval and Noctor (2007). In short, ascorbic acid and its oxidized form, dehydroascorbic acid were extracted with 6% *m*-phosphoric acid (w/v) and 0.2 mM diethylenetriaminepentaacetic acid, using ultrasonication and vortexing. After centrifugation at 600 *g* for 10 min at 4°C, the supernatants were collected and the pellet was re-extracted following the same procedure. Their levels were determined spectrophotometrically at 265 nm, using the ascorbate oxidase assay. The oxidized state of ascorbate was calculated as DHA/(AA + DHA) × 100, where AA is ascorbate and DHA is dehydroascorbate.

The analysis of vitamin E was performed as described (Amaral et al., 2005). In short, 200 mg per sample were extracted with methanol, exactly as described for anthocyanins, and then filtered prior to HPLC analyses. The HPLC equipment consisted of an integrated system with a Jasco PU-2089 Plus pump, a Jasco AS-2055 Plus auto-sampler and a FP-1520 fluorescence detector (Jasco, Tokyo, Japan). All tocopherol and tocotrienol forms were separated on an Inertsil 100A (5 μm, 30 × 250 mm, GL Sciences Inc., Tokyo, Japan) normal-phase column, operating at room temperature. The flow rate was 0.7 mL min^-1^ and the injection volume was 10 μL. The mobile phase was a mixture of *n*-hexane and *p*-dioxane (95.5:4.5, v/v). Detection was carried out at an excitation of 295 nm and emission at 330 nm. Quantification was based on the results obtained from the fluorescence signal and compared to that of a calibration curve made with authentic standards of each compound (Sigma–Aldrich, Steinheim, Germany).

### Statistical Analysis

Data were analyzed by using one-way (first experiment) or two-way (second experiment) factorial analysis of variance (ANOVA). Multiple comparisons tests were carried out by using Bonferroni *post-hoc* tests. In all cases, differences were considered significant at a probability level of *P* ≤ 0.05. Furthermore, correlation analyses using the Spearman rank’s correlation were made. All statistical analyses were performed using the SPSS 20.0 statistical package.

## Results

### ABA Levels Increase During Ripening on the Tree but Decrease During Over-Ripening

**FIGURE 1:**
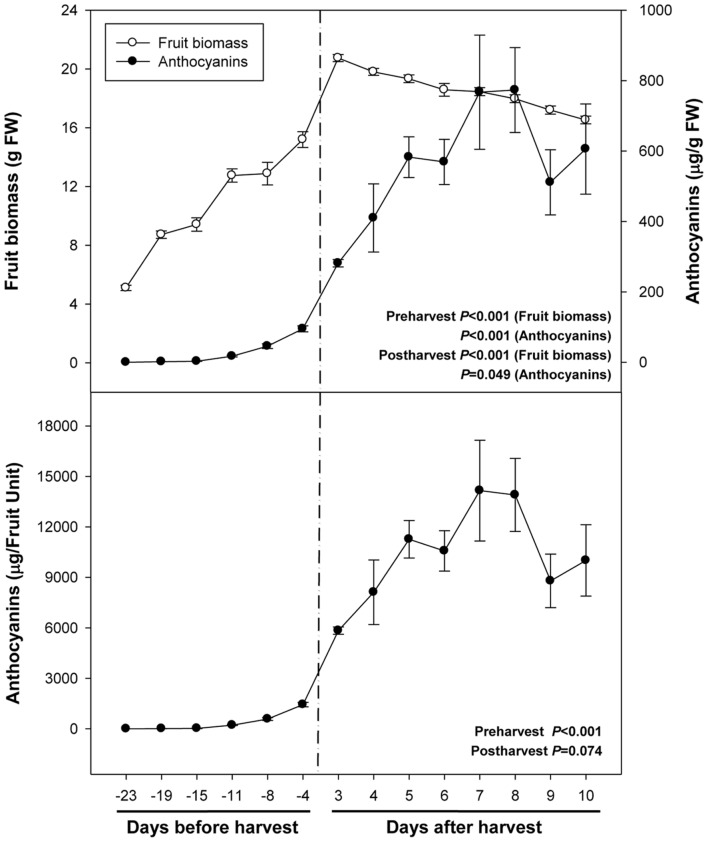
**Fruit biomass and levels of total anthocyanins during ripening on the tree (pre-harvest) and during over-ripening at 23°C (post-harvest).** Data are the mean ± SE of *n* = 8 (pre-harvest) and *n* = 3 (post-harvest) for anthocyanins, and *n* = 8 (pre-harvest) and *n* = 6 (post-harvest) for fruit biomass. Statistical analyses were performed by one-way ANOVA to test for the effects of time during pre- and post-harvest. Results of statistics are shown in the inlets. Differences were considered significant when *P* ≤ 0.05. NS, not significant. Anthocyanin levels are given both per fresh weight (FW) and per fruit unit. Harvest (time 0 in the *X* axis) corresponds to 57 days after full bloom.

Fruit biomass increased fivefold during ripening on the tree (from 23 days pre-harvest to 3 days post-harvest), to decrease later by 20% due to over-ripening for 1 week (from day 3 to day 10 of post-harvest at 23°C, **Figure 1**). Anthocyanin levels increased from non-detectable values to 95 μg/g fruit during pre-harvest (between 23 and 4 days preharvesst), to increase even further up to 582 μg/g fruit at 5 days post-harvest. Then, anthocyanin levels remained relatively constant at high levels during over-ripening until the end of the experiment (10 days post-harvest, **Figure 1**).

**FIGURE 2:**
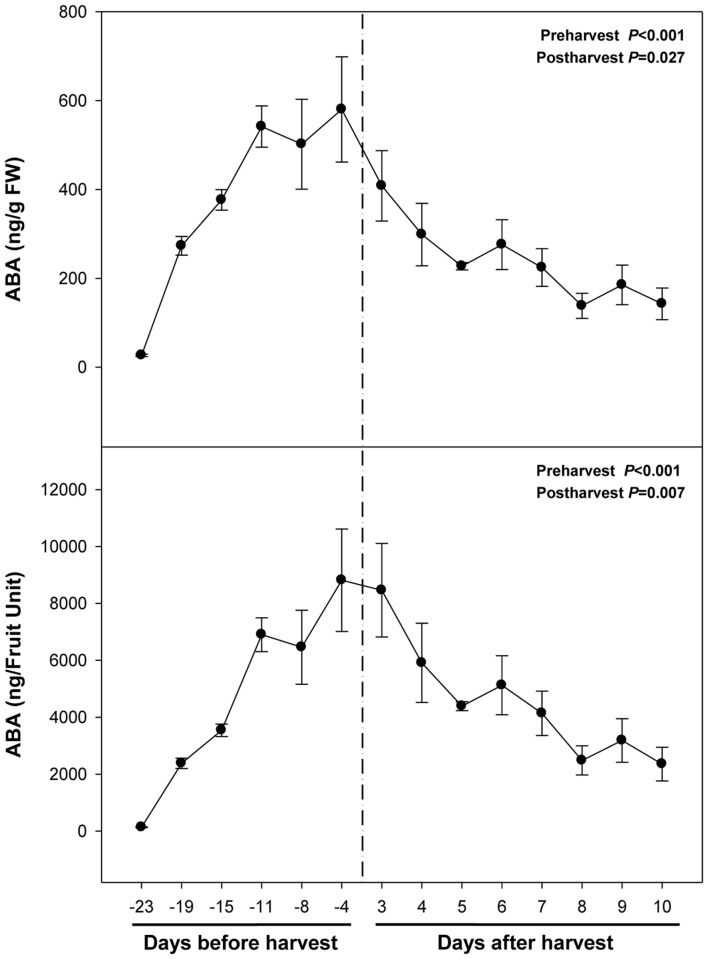
**Endogenous concentrations of abscisic acid (ABA) during ripening on the tree (pre-harvest) and during over-ripening at 23°C (post-harvest).** Data are the mean ± SE of *n* = 8 (pre-harvest) and *n* = 3 (post-harvest). Statistical analyses were performed by one-way ANOVA to test for the effects of time during pre- and post-harvest. Results of statistics are shown in the inlets. Differences were considered significant when *P* ≤ 0.05. NS, not significant. ABA levels are given both per FW and per fruit unit. Harvest (time 0 in the *X* axis) corresponds to 57 days after full bloom.

Abscisic acid levels increased sharply from 26 ng/g fruit at 23 days pre-harvest to 540 ng/g fruit at 11 days pre-harvest, to keep later constant until 4 days pre-harvest (**Figure 2**). Over-ripening at 23°C led to a depletion of endogenous ABA concentrations in the fruit to attain minimum values of 142 ng/g fruit at 10 days post-harvest. It is noteworthy that ABA increases preceded anthocyanin accumulation during pre-harvest. In contrast, ABA did not change in parallel with anthocyanin accumulation during post-harvest (**Figures 1** and **2**).

Levels of carotenoids decreased sharply during fruit ripening on the tree (**Table 1**). Violaxanthin, an ABA precursor, decreased from 0.58 mg/g FW at 23 days to non-detectable values at 4 days pre-harvest (**Table 1**), which occurred in parallel with increases of ABA levels during fruit ripening on the trees (**Figure 2**). Lutein and zeaxanthin levels also decreased progressively down to non-detectable values during fruit ripening on the tree, while fruits at 4 days pre-harvest still kept 0.17 mg/g FW of β-carotene. The amounts of this antioxidant expressed per fruit unit increased during ripening, attaining maximum levels of 3 mg per fruit unit at 4 days pre-harvest (**Table 1**). Carotenoids were not detected during post-harvest (data not shown).

**Table 1:** Carotenoid levels during sweet cherry ripening on the tree.

Days pre-harvest	Violaxanthin	Lutein	Zeaxanthin	β-carotene
**Carotenoids (mg/g FW)**				
23	0.58 ± 0.18^a^	3.35 ± 0.25^a^	0.43 ± 0.05^a^	0.49 ± 0.10^a^
15	0.14 ± 0.03^b^	0.73 ± 0.11^b^	0.29 ± 0.05^a^	0.20 ± 0.06^ab^
4	ND^b^	ND^c^	ND^b^	0.17 ± 0.08^b^
**Carotenoids (mg/g Fruit unit)**				
23	0.47 ± 0.15^a^	2.74 ± 0.20^a^	0.35 ± 0.04^a^	0.40 ± 0.08^a^
15	1.34 ± 0.30^b^	6.86 ± 1.04^b^	2.74 ± 0.47^b^	1.65 ± 0.75^ab^
4	ND^a^	ND^c^	ND^a^	3.06 ± 0.91^b^

*Levels of violaxanthin, lutein, zeaxanthin and β-carotene are given at 23, 15, and 4 days pre-harvest, both on a fresh weight (FW) and fruit unit basis. ND, not detected. Different letters indicate significant differences between time points using Bonferroni post hoc tests (ANOVA, P < 0.05).*

**FIGURE 3:**
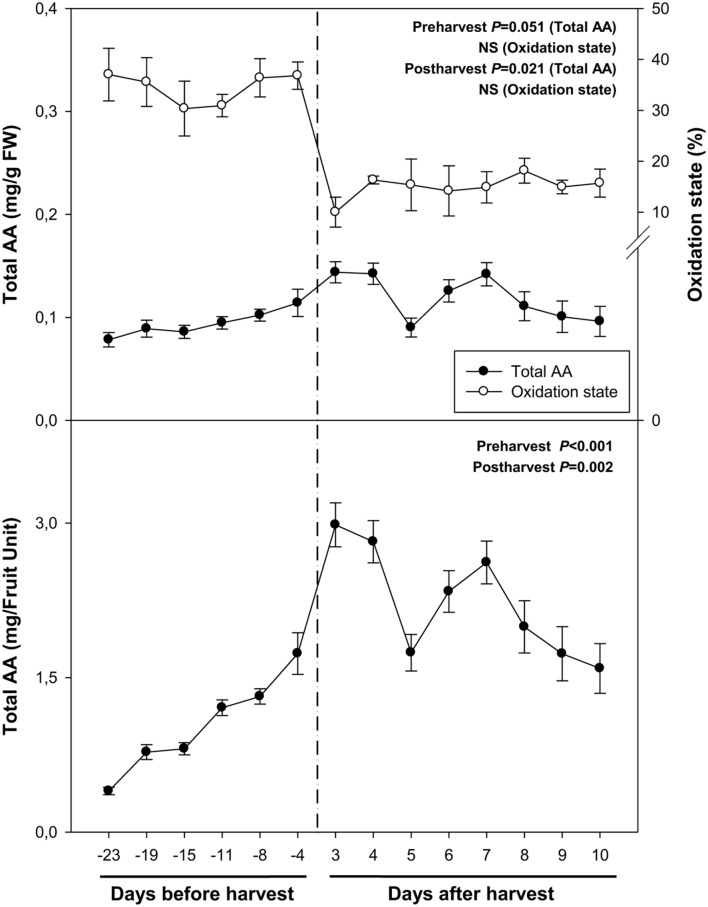
**Total ascorbate (AA) and its oxidation state during ripening on the tree (pre-harvest) and during over-ripening at 23°C (post-harvest).** Data are the mean ± SE of *n* = 8 (pre-harvest) and *n* = 3 (post-harvest). Statistical analyses were performed by one-way ANOVA to test for the effects of time during pre- and post-harvest. Results of statistics are shown in the inlets. Differences were considered significant when *P* ≤ 0.05. NS, not significant. AA levels are given both per FW and per fruit unit. Oxidation state was calculated as oxidized ascorbate per total ascorbate. Harvest (time 0 in the *X* axis) corresponds to 57 days after full bloom.

Total ascorbate levels increased during pre-harvest to decrease later during post-harvest, both when expressed on a fresh weight and a fruit unit basis (**Figure 3**). Interestingly, ascorbate levels showed a biphasic response during post-harvest, with minimum ascorbate levels at 5 and 10 days post-harvest. It is noteworthy that the oxidation state of ascorbate kept constant, both, during pre- and post-harvest, but decreased sharply from around 40% to levels below 20% just after harvest (**Figure 3**).

**FIGURE 4:**
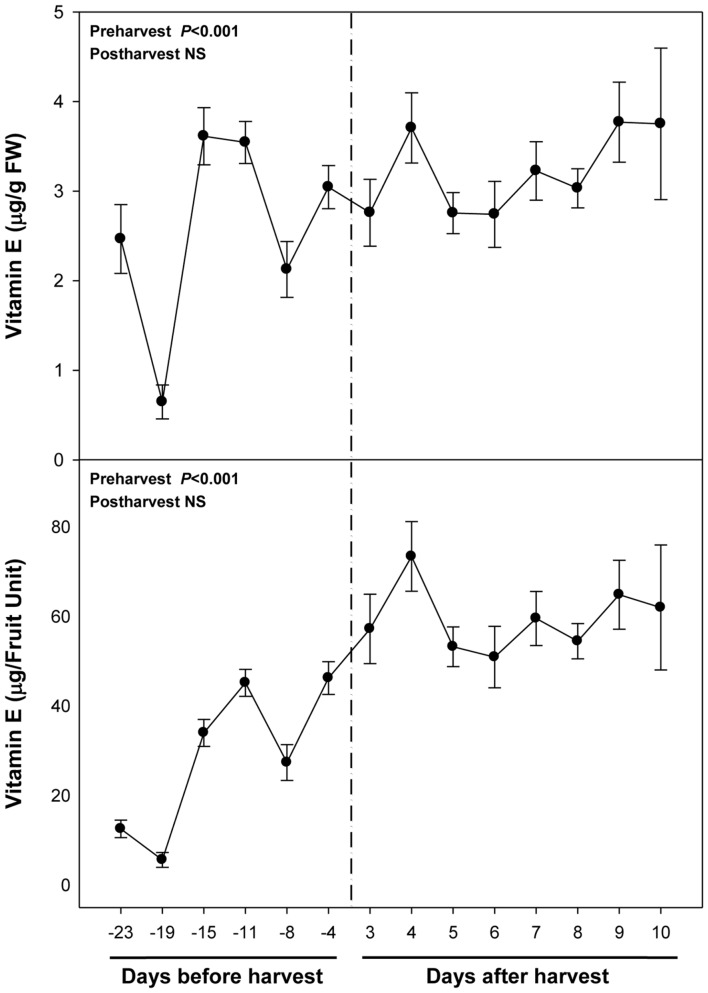
**Total vitamin E levels during ripening on the tree (pre-harvest) and during over-ripening at 23°C (post-harvest).** Data are the mean ± SE of *n* = 8 (pre-harvest) and *n* = 3 (post-harvest). Statistical analyses were performed by one-way ANOVA to test for the effects of time during pre- and post-harvest. Results of statistics are shown in the inlets. Differences were considered significant when *P* ≤ 0.05. NS, not significant. Vitamin E levels are given both per FW and per fruit unit. Harvest (time 0 in the *X* axis) corresponds to 57 days after full bloom.

Vitamin E levels were much lower than those of ascorbate, with maximum levels of 3.5 μg/g fruit being attained at 15 and 11 days pre-harvest and at the end of the experiment (**Figure 4**). Sharp fluctuations in total vitamin E levels were mainly due to those of α-tocopherol, the major vitamin E form present in fruits (Supplementary Figure S1). γ-Tocopherol levels were lower but also more stable than those of α-tocopherol. Vitamin E levels tended to increase during ripening, an effect that was particularly observed for γ-tocopherol (Supplementary Figure S1) and when results where expressed on a fruit unit basis (**Figure 4**). Neither vitamin E levels nor those of α- and γ-tocopherol were altered during post-harvest either when expressed per g fruit or per fruit unit (**Figure 4**; Supplementary Figure S2). β- and δ-tocopherols, and tocotrienols were not detected in cherry fruits.

### Variations in ABA Levels during Cold Storage

**FIGURE 5:**
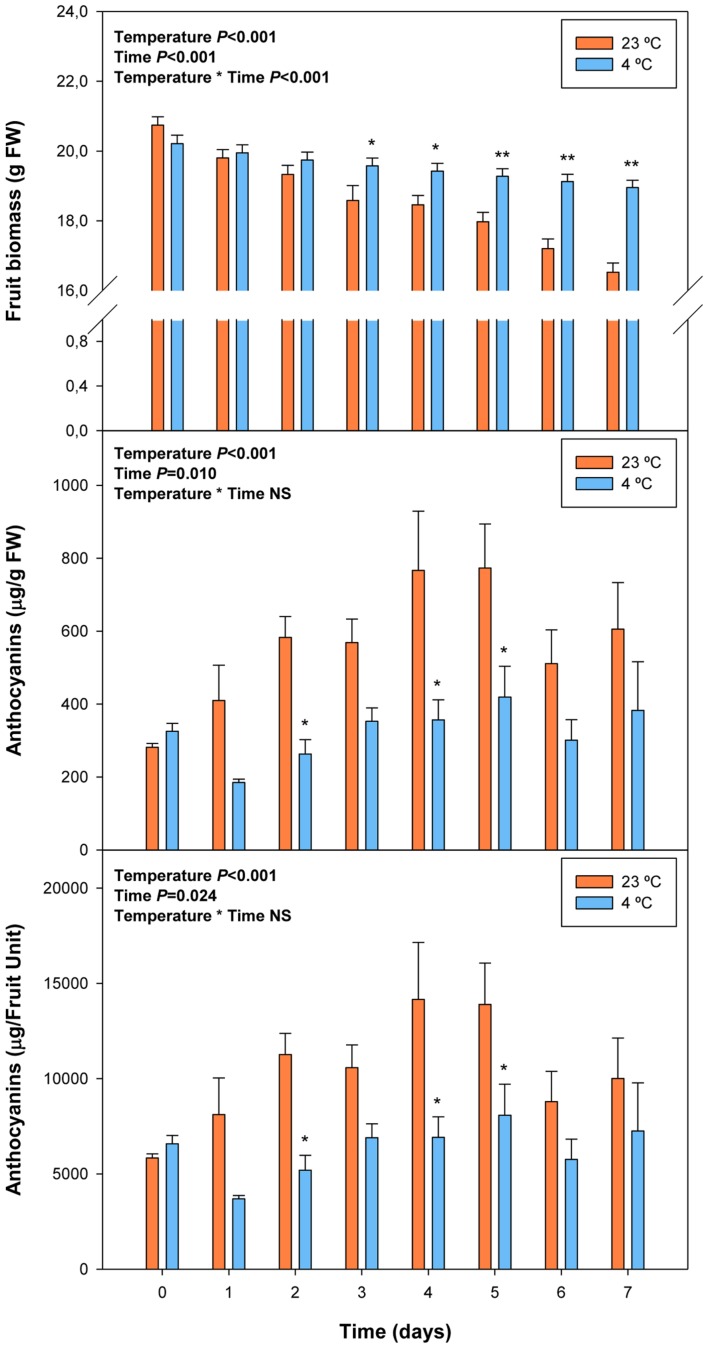
**Effects of cold storage on fruit biomass and levels of total anthocyanins during post-harvest.** Data are the mean ± SE of *n* = 3 for anthocyanins and *n* = 6 for fruit biomass. Statistical analyses were performed by two-way ANOVA to test for the effects of treatment and time. Results of statistics are shown in the inlets. One or two asterisks are shown when differences between treatments are significant or highly significant (*P* ≤ 0.05 and 0.001, respectively, Bonferroni *post hoc* test) at any given time point. NS, not significant. Anthocyanin levels are given both per FW and per fruit unit.

**FIGURE 6:**
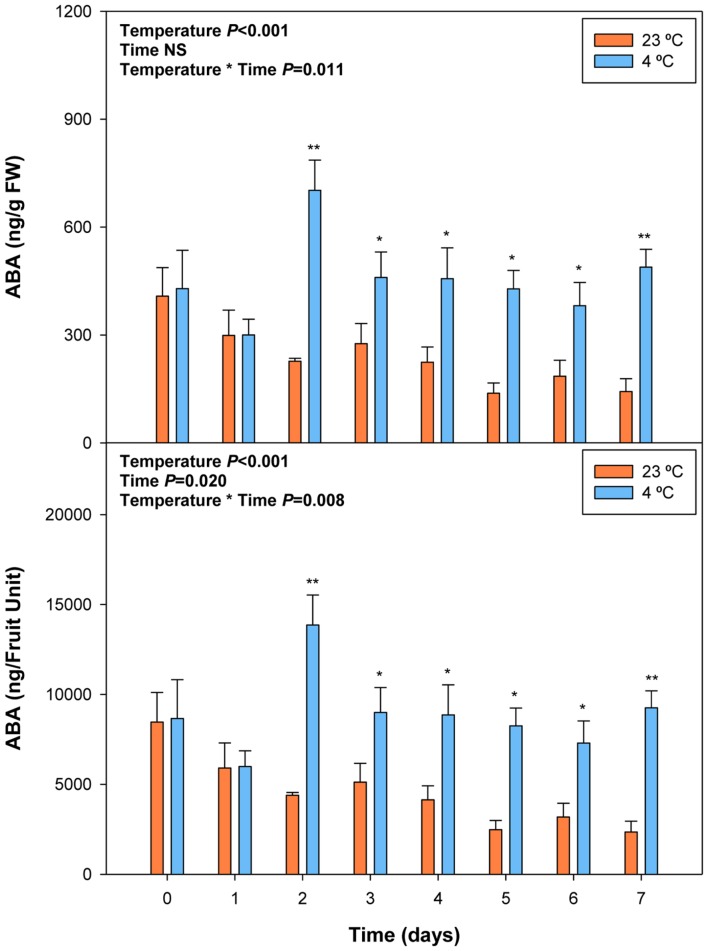
**Effects of cold storage on the endogenous concentrations of ABA during post-harvest.** Data are the mean ± SE of *n* = 3. Statistical analyses were performed by two-way ANOVA to test for the effects of treatment and time. Results of statistics are shown in the inlets. One or two asterisks are shown when differences between treatments are significant or highly significant (*P* ≤ 0.05 and 0.001, respectively, Bonferroni *post hoc* test) at any given time point. NS, not significant. ABA levels are given both per FW and per fruit unit.

Cold storage prevented over-ripening, as observed with the maintenance of visual fruit firmness (Supplementary Figure S2), biomass and anthocyanin levels (**Figure 5**). Cold treatment prevented anthocyanin accumulation, an effect that was already observed at 2 days of cold storage. ABA levels increased in response to cold storage, with an increment at 2 days of treatment (**Figure 6**). Thereafter, ABA levels in cold-stored fruits did not increase further but kept always at higher levels compared to fruits stored at 23°C. In this case, ABA levels inversely correlated, or simply did not correlate with those of anthocyanins. During over-ripening at 23°C, ABA levels decreased, while those of anthocyanins increased. When over-ripening was prevented by cold storage, enhanced ABA levels did not lead to changes in anthocyanin accumulation.

**FIGURE 7:**
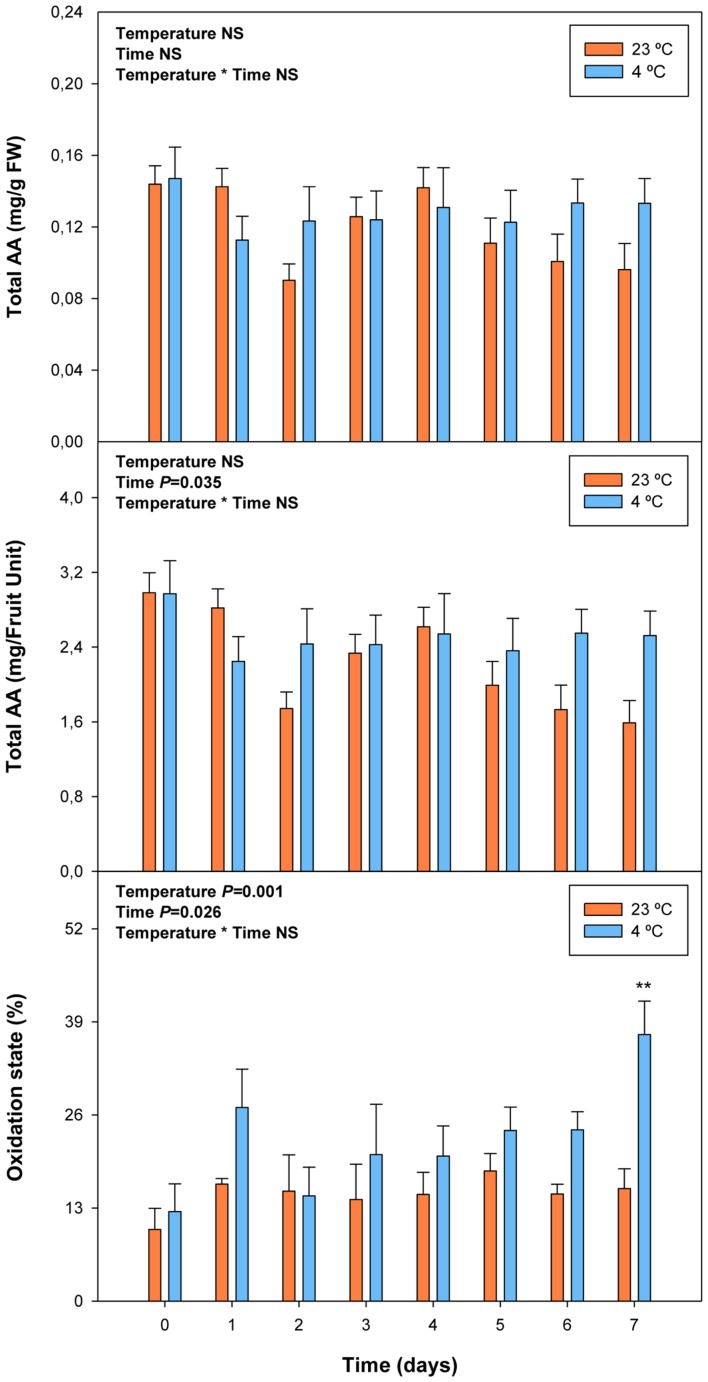
**Effects of cold storage on total ascorbate (AA) and its oxidation state during post-harvest.** Data are the mean ± SE of *n* = 3. Statistical analyses were performed by two-way ANOVA to test for the effects of treatment and time. One or two asterisks are shown when differences between treatments are significant or highly significant (*P* < 0.05 and 0.001, respectively, Bonferroni *post hoc* test). Results of statistics are shown in the inlets. Differences were considered significant when *P* ≤ 0.05. NS, not significant. AA levels are given both per FW and per fruit unit. Oxidation state was calculated as oxidized ascorbate per total ascorbate.

**FIGURE 8:**
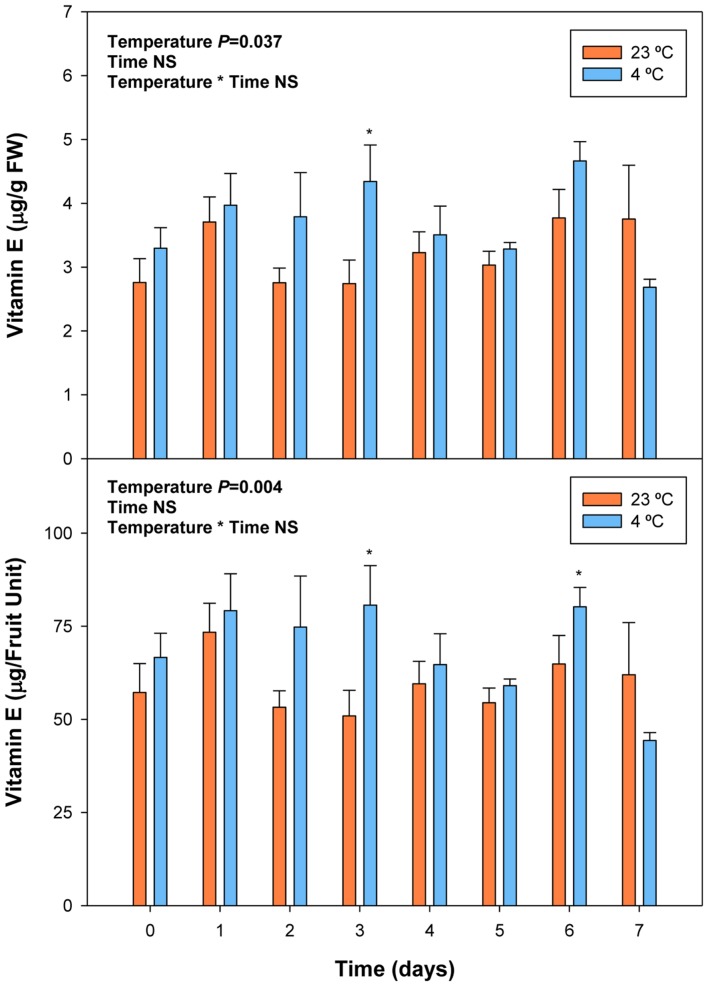
**Effects of cold storage on total vitamin E levels during post-harvest.** Data are the mean ± SE of *n* = 3. Statistical analyses were performed by two-way ANOVA to test for the effects of treatment and time. Results of statistics are shown in the inlets. One or two asterisks are shown when differences between treatments are significant or highly significant (*P* ≤ 0.05 and 0.001, respectively, Bonferroni *post hoc* test) at any given time point. NS, not significant. Vitamin E levels are given both per FW and per fruit unit.

**FIGURE 9:**
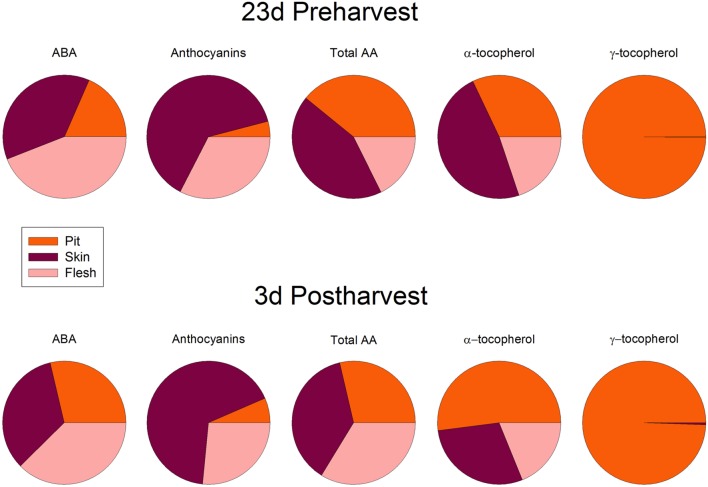
**Abscisic acid, anthocyanins, total ascorbate (AA) and levels of α- and γ-tocopherol in the pit, skin and flesh of sweet cherries.** Data are the mean ± SE of *n* = 4. Results are given as a percentage after calculating the levels of each compound per FW in every tissue. All standard errors were below 10% in all cases.

Cold storage did not alter total ascorbate levels, but affected its oxidation state. The ascorbate oxidation state increased in response to cold treatment, but differences were small and *post hoc* analyses did not reveal significant difference at any time point (**Figure 7**). In contrast, ABA levels correlated with vitamin E levels in cold-stored fruits, those of total vitamin E were increasing in parallel with ABA, during the first days of cold treatment (**Figure 8**). The levels of α- and γ-tocopherol were not significantly altered by cold treatment when analyzed separately (Supplementary Figure S3), thus indicating that cold effects on total vitamin E levels (**Figure 8**) were cumulative. It is noteworthy that α- and γ-tocopherol followed a completely different tissue-specific accumulation, with γ-tocopherol accumulating, almost exclusively (>99%), in the pit (**Figure 9**). In contrast, α-tocopherol, anthocyanins, ascorbate and ABA were all detected in the pit, flesh and skin during both pre- and post-harvest (**Figure 9**).

## Discussion

Sweet cherry is a non-climacteric fruit, which ripening is known to be promoted by ABA (Setha et al., 2005). Its ethylene concentration is low and has no direct effect in the ripening of sweet cherries (Hartmann, 1992; Kondo and Gemma, 1993), although it may influence anthocyanin accumulation (Kondo and Inoue, 1997). ABA is, however, the phytohormone that plays a major role in the regulation of anthocyanin accumulation and organoleptic sweet cherries properties, such as the ratio of total soluble sugars to total acidity (Kondo and Gemma, 1993; Kondo and Inoue, 1997; Luo et al., 2013). Studies in other non-climacteric fruits, such as grapes, have also shown that ABA not only modulates color development and sugar accumulation, but it may also be implicated in the control of softening during the ripening process (Castellarin et al., 2016). Here, we provide correlative evidence supporting a role for ABA in the regulation of both anthocyanin and vitamin E accumulation during pre-harvest, but not during post-harvest, in sweet cherries “Prime Giant.” Furthermore, results suggest that ABA may help prevent over-ripening during post-harvest at 4°C.

We found that ABA levels strongly and positively correlate with anthocyanin accumulation during ripening of fruits on the tree (**Table 2**), which is in agreement with previous studies (Luo et al., 2013). However, a strong negative correlation was observed between endogenous concentrations of ABA and anthocyanin levels during post-harvest (**Table 2**). Over-ripening at 23°C led to progressive decreases in ABA concentrations, while anthocyanin accumulation kept at high levels, thus suggesting an inhibitory role for ABA in over-ripening (**Figures 1** and **2**). Furthermore, ABA levels increased after 2 days of cold storage, while anthocyanin levels kept at lower levels at 4°C relative to 23°C (**Figures 5** and **6**), thus suggesting ABA might prevent over-ripening in cold-stored fruits. The role of ABA in over-ripening has been poorly studied to date, particularly in non-climacteric fruits. However, the application of antitranspirants, such as ABA, in rambutan, a non-climacteric fruit, has been shown to be effective in preventing over-ripening (Siriphollakul et al., 2006), thus supporting further the idea that ABA helps promote ripening in fruits on the tree, but delays over-ripening in detached fruits during post-harvest. This indicates that ABA does not act alone but together with other signaling compounds in the regulation of the ripening process in fruits on the tree, an aspect that warrants further investigations.

**Table 2:** Results of Spearman’s rank correlation analyses between endogenous concentrations of ABA and quality parameters during pre- and post-harvest.

Parameters	Experiments 1 and 2 (All data)	Experiment 1	Pre-harvest	Post-harvest	Experiment 2
Anthocyanins	0.009	0.033	**0.703^∗∗^**	-0.354	-**0.531^∗∗^**
Total AA	0.147	0.064	0.219	0.290	0.239
Oxidation state	0.132	0.169	-0.133	-0.287	0.251
Vitamin E	0.196^∗^	0.124	0.282^∗^	-0.137	0.130
α-tocopherol	0.155	0.041	0.176	-0.046	0.147
γ-tocopherol	0.186	0.180	**0.367^∗^**	-0.179	-0.001

*Correlation coefficients values are given followed by one or two asterisks when the correlation is significant or highly significant (P ≤ 0.05 and 0.001, respectively). Significant correlations with coefficients above 0.35 are shown in bold. Absence of an asterisk indicates the correlation was not significant. Data was analyzed altogether, and also separately (for each experiment, differentiating also pre- and post-harvest data for Experiment 1).*

Aside from its role in the regulation of the ripening process of fruits on the tree, by modulating softening, sugar accumulation and color development (though the modulation of anthocyanin accumulation, Luo et al., 2013; Wang et al., 2015; Castellarin et al., 2016), nothing is known about the possible effects of ABA on vitamin accumulation in sweet cherries or other non-climacteric fruits. Vitamin C is a water-soluble compound that acts as a cofactor for many iron and copper hydroxylases and dioxygenases involved in key physiological processes in humans, such as in the production of collagen and the synthesis of carnitine (Bender, 2003; Johnston et al., 2007). That is the reason why the frequent intake of foods rich in bioactive compounds, such as vitamin C, is associated with a healthy diet (Arrigoni and De Tullio, 2002). In addition, this compound is considered as one of the most important antioxidants for plant growth and defense (Foyer and Noctor, 2011), which is present in many plant cell compartments, such as mitochondria, plastids, peroxisomes and the apoplast (Smirnoff, 2000; Foyer, 2001). Moreover, ascorbate is the principal non-enzymatic water-soluble antioxidant that is able to eliminate reactive oxygen species (Cadenas and Packer, 2002). Vitamin C is especially vulnerable to oxidative and enzymatic degradation in raw fruits and vegetables (Redmond et al., 2003). Some studies have reported a loss of vitamin C in many fruits stored under non-optimal conditions after harvest (Munyaka et al., 2010; Neves et al., 2015). Although correlation analyses did not reveal any significant relationship between endogenous concentrations of ABA and vitamin C levels during ripening, over-ripening or cold treatment (**Table 2**), the present study confirmed that this vitamin is present at high amounts in sweet cherries, attaining maximum levels of 3 mg per fruit unit just after harvest, and it was found that its oxidation increases after 7 days of cold storage. Furthermore, ascorbate is known to act as a cofactor of 9-*cis*-epoxycarotenoid dioxygenase (NCED), the key limiting step in the biosynthesis of ABA from carotenoids, particularly neoxanthin and/or violaxanthin (Conklin and Barth, 2004). In the present study, violaxanthin levels decreased concomitantly with increases of ABA levels during ripening of fruits on the trees, which is consistent with a role for violaxanthin as a precursor of ABA in sweet cherries (Luo et al., 2013).

On the other hand, vitamin E, a lipid-soluble antioxidant in cell membranes, also with health-promoting effects (Booth et al., 2004), is found at high concentrations in some fruits, such as kiwis or avocados (Chun et al., 2006), but it has received little consideration in sweet cherries, mainly due to their low levels in the fruit, at least, compared to other antioxidants, such as anthocyanins or vitamin C. Among vitamin E compounds, both α- and β-tocopherol were previously shown to be present in sweet cherries, being α-tocopherol the most abundant with amounts around 1 μg/g fruit (Bastos et al., 2015), which is similar to the amounts obtained in the present study (Supplementary Figure S3). However, we did not detect β- but, instead, γ-tocopherol in sweet cherries, which accumulated particularly in the pit (**Figure 9**). Most importantly, we found a positive correlation between endogenous concentrations of ABA and vitamin E accumulation in sweet cherries, particularly at pre-harvest (**Table 2**). Interestingly, endogenous concentrations of ABA correlated more strongly with γ- than with α-tocopherol levels. Previous studies have shown the presence of an ABA-responsive element (ABRE) in the promoter region of *HYDROXYPHENYLPYRUVATE DIOXYGENASE* (*HPPD*), which encodes for the enzyme responsible of the formation of homogentisate, needed for the biosynthesis of all vitamin E compounds (Chaudhary and Khurana, 2009; Falk and Munné-Bosch, 2010). Therefore, our data supports the contention that ABA is implicated in the biosynthesis of vitamin E compounds in sweet cherries, as it has been shown in leaves of plants exposed to various abiotic stresses (Chaudhary and Khurana, 2009; Munné-Bosch et al., 2009). It is noteworthy that the correlative evidence obtained in the present study supporting a link between ABA and vitamin E biosynthesis was observed in fruits that were ripening on the tree during pre-harvest, but not during post-harvest at 23°C. Furthermore, enhanced vitamin E levels were preceded by ABA increases during cold storage, thus suggesting ABA may also regulate tocopherol accumulation in response to cold stress in sweet cherries. This may indeed be a defensive response, since both ABA and tocopherols are known to be needed to combat cold-induced reactive oxygen production in plants (El Kayal et al., 2006).

## Conclusion

The ABA plays a major role in the control of the ripening process in sweet cherries, particularly stimulating this process during pre-harvest and positively influencing quality parameters, such as the accumulation of anthocyanins and vitamin E. Further research is, however, needed to better understand the mechanisms underlying the regulation of vitamin E biosynthesis by ABA during pre-harvest and cold storage, as well as the inhibitory role of ABA in the over-ripening of sweet cherries, beyond its possible function as an antitranspirant.

## Author Contributions

VT and SM-B conceived and designed the experiments with the help of NT. VT, NT, and PM performed the experiments. SM-B wrote the manuscript with the help of VT; all authors contributed to the discussion, revised and approved the final manuscript.

## Conflict of Interest Statement

The authors declare that the research was conducted in the absence of any commercial or financial relationships that could be construed as a potential conflict of interest.
