# Supplementary material for: Implication of Abscisic Acid on Ripening and Quality in Sweet Cherries: Differential Effects during Pre- and Post-harvest
Source: Front Plant Sci. 2016 May 4;7:602. doi: 10.3389/fpls.2016.00602 (PMC4855249; doi:10.3389/fpls.2016.00602)
Supplement: Supplementary file 1 [file Data_Sheet_1.DOCX]

**Supplementary Information**

**Suppl. Figure 1. Levels of α- and γ-tocopherols during ripening on the tree (preharvest) and during over-ripening at 23ºC (postharvest).** Data are the mean ± SE of n=8 (preharvest) and n=3 (postharvest). Statistical analyses were performed by one-way ANOVA to test for the effects of time during pre- and postharvest. Results of statistics are shown in the inlets. Differences were considered significant when *P*≤0.05. NS, not significant. Vitamin E levels are given both per fresh weight (FW) and per fruit unit.

**Suppl. Figure 2. Cold storage improves firmness in sweet cherries.** Photographs of fruits stored at 23 ºC and 4 ºC were taken after 7d and 10d postharvest.


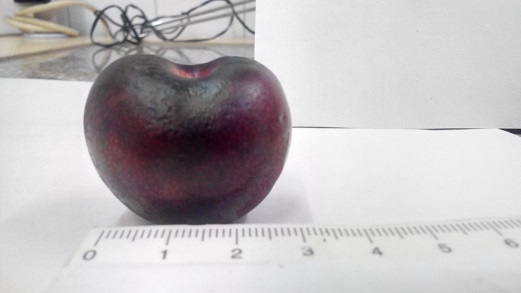


**4 ºC, 10d**


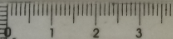

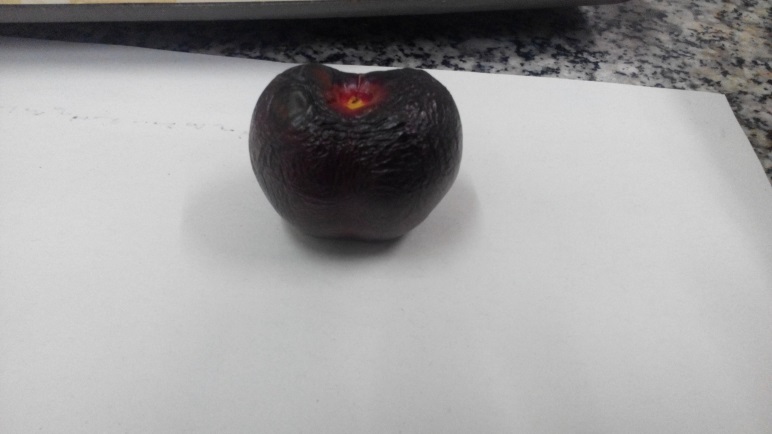


**23 ºC, 7d**


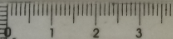

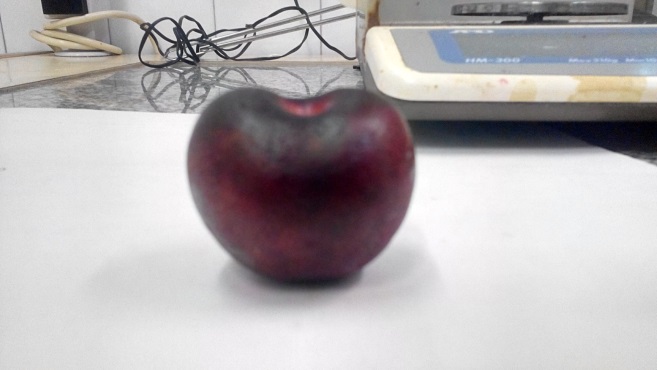


**4 ºC, 7d**

**23 ºC, 10d**


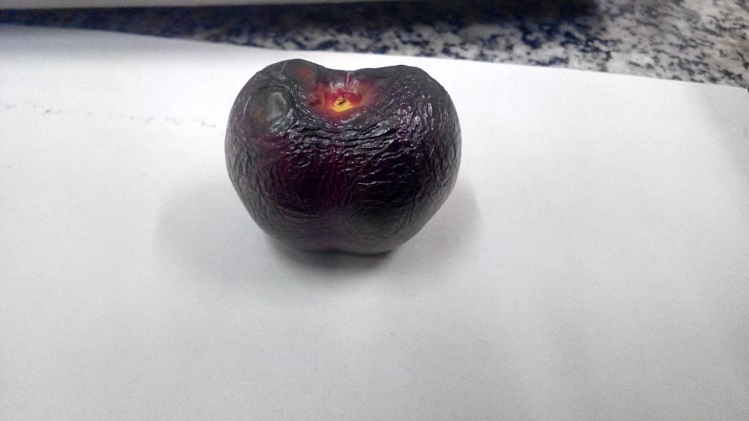


**Suppl. Figure 3. Cold treatment effects on the levels of α- and γ-tocopherols during postharvest storage of sweet cherries.** Data are the mean ± SE of n=3. Statistical comparisons were performed by two-way ANOVA.NS, not significant.
